# Common Interactions between S100A4 and S100A9 Defined by a Novel Chemical Probe

**DOI:** 10.1371/journal.pone.0063012

**Published:** 2013-05-08

**Authors:** Per Björk, Eva Källberg, Ulf Wellmar, Matteo Riva, Anders Olsson, Zhifei He, Marie Törngren, David Liberg, Fredrik Ivars, Tomas Leanderson

**Affiliations:** 1 Immunology Group, Lund University, Lund, Sweden; 2 Active Biotech AB, Lund, Sweden; National Cancer Institute at Frederick, United States of America

## Abstract

S100A4 and S100A9 proteins have been described as playing roles in the control of tumor growth and metastasis. We show here that a chemical probe, oxyclozanide (OX), selected for inhibiting the interaction between S100A9 and the receptor for advanced glycation end-products (RAGE) interacts with both S100A9 and S100A4. Furthermore, we show that S100A9 and S100A4 interact with RAGE and TLR4; interactions that can be inhibited by OX. Hence, S100A4 and S100A9 display similar functional elements despite their primary sequence diversity. This was further confirmed by showing that S100A4 and S100A9 dimerize both *in vitro* and *in vivo*. All of these interactions required levels of Zn^++^ that are found in the extracellular space but not intracellularly. Interestingly, S100A4 and S100A9 are expressed by distinct CD11b^+^ subpopulations both in healthy animals and in animals with either inflammatory disease or tumor burden. The functions of S100A9 and S100A4 described in this paper, including heterodimerization, may therefore reflect S100A9 and S100A4 that are released into the extra-cellular milieu.

## Introduction

S100 proteins constitute a protein family with more than 20 members, many of which have been associated with various diseases [1]. Interestingly, while seemingly divergent with regard to their primary sequence, the three-dimensional structure of most of the members is very similar [2], indicating that there might be shared functions between the protein family members.

S100A4, also called metastasin, has been shown to promote tumor growth and metastasis in several tumor models [3]–[5]. S100A4 has also been shown to be involved in the control of tumor growth in human cancer [6], and to be a potential prognostic marker [7]–[9]. S100 proteins can have both intra-cellular and extra-cellular functions and S100A4 has been shown to have a role in both compartments. The S100A4 protein is directly involved in the expression of the tissue degrading matrix metalloprotease MMP-13, both by interaction of extra-cellular S100A4 with RAGE [10], but also via a direct involvement in transcription of the MMP-13 gene [11]. S100A4 has also been shown to be involved in the regulation of cell motility [12] and angiogenesis [13].

S100A9 is a protein that is highly expressed in granulocytes but also expressed in some monocytic subpopulations [14]. S100A9 protein is present in plasma at microgram levels in the form of heterodimers together with S100A8 [15]. Furthermore, the S100A8/A9 protein level in plasma can be greatly increased in patients with inflammatory disease [16], or malignancies [17]. S100A9 can also be expressed as a homodimer in the absence of S100A8 [18].

With regard to biological function, S100A9 has been ascribed both intracellular and extracellular functions. S100A9 can be phosphorylated by p38 MAPK which in turn regulates its binding to the cytoskeleton [19], indicating that S100A9 may be involved in regulation of cell motility [20]. S100A9 has also been shown to be a ligand for two pro-inflammatory extra-cellular receptors, RAGE and TLR4 [21]. Interestingly, both RAGE and TLR4 have been shown to play a role in the control of tumor growth in different systems [22], [23], and it has also been shown that inhibition of S100A9/TLR4 interactions can inhibit tumor growth [24]. S100A9 may also be involved in cancer progression by separate mechanisms. It has been demonstrated that S100A9 is important for metastasis, most likely by interfering at an early stage of metastasis formation [25]. Furthermore, treatment with an S100A9-binding small molecule will inhibit metastasis formation in a prostate cancer tumor model [26]. Lastly, S100A9 has also been shown to be important for the development and function of so called myeloid derived suppressor cells (MDSC) [27], [28]. Thus, S100A9 is emerging as a potential pharmaceutical target for the treatment of malignant disease.

In this study we identify a chemical probe that inhibits the interaction between S100A9 and RAGE or TLR4. We could show that this molecule also inhibited tumor growth *in vivo*. Interestingly, the same chemical probe also bound and inhibited S100A4 interactions with the same receptors. Hence, our findings demonstrate that it is feasible to develop small molecule inhibitors that can bind multiple S100 proteins and inhibit their interaction with biologically relevant receptors.

## Results

### Validating S100A9 as a Pharmaceutical Target Using a Chemical Probe

**Figure 1:**
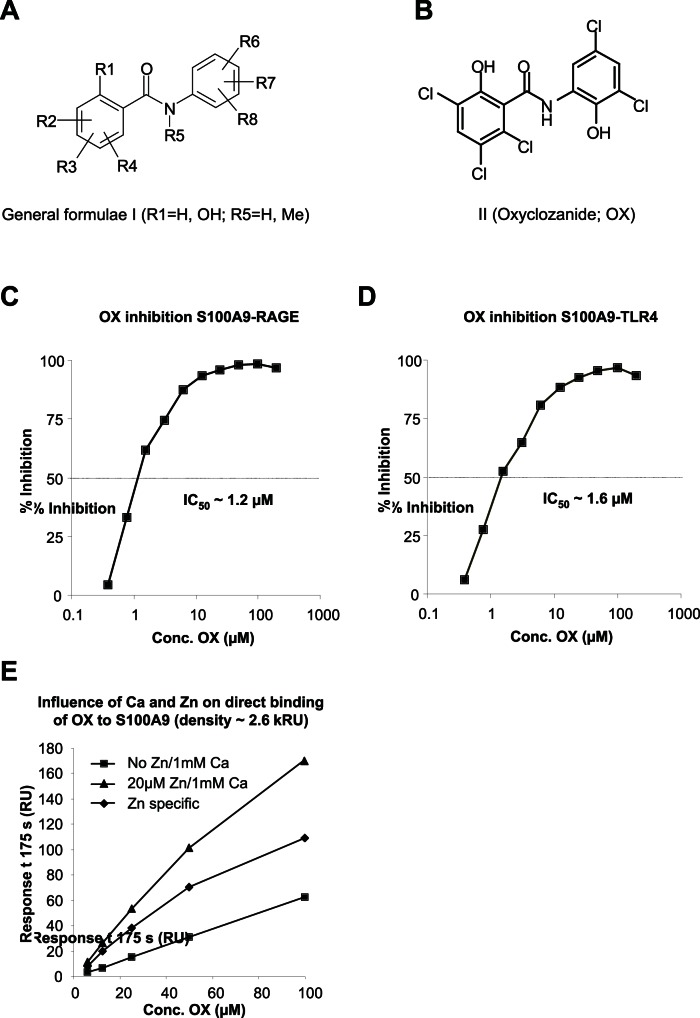
Oxyclozanide binds S100A9 protein. A: General formulae of synthesized salicylic and benzoic amides. B: Structure of Oxyclozanide (OX). C-D: Inhibition of S100A9 binding to immobilized RAGE (C) or TLR4 (D) by OX. 50 nM S100A9 was injected over RAGE or TLR4±0.4–200 µM OX in the presence of 1 mM CaCl_2_, 20 µM ZnCl_2_ and 1% DMSO. Binding was expressed as % inhibition of S100A9 response without OX and IC_50_ calculated after curve fit to a sigmoidal dose-response model. IC_50_ values of ∼1.3 and 1.6 µM were obtained for OX inhibition of S100A9 binding to RAGE and TLR4. E: Effect of Zn^++^ on direct binding of OX to amine coupled S100A9. 3.125–100 µM OX was injected (2 min at 30 µL/min) over S100A9 (density ∼2.6 kRU) in HBS-P with 1 mM Ca^++^ ±20 µM Zn^++^. Responses at late association phase are plotted versus OX concentration and curves fit to a 1∶1 model. Zn^++^ specific binding was obtained by subtraction of responses with Ca^++^ alone.

Given our previous interest in S100A9, and in particular its interactions with RAGE and TLR4 [21], [24], we decided to screen an in house chemical library for new molecules that bind to S100A9 and inhibit its interaction with RAGE and TLR4. Early on in this screen we identified salicylic amides (Figure 1A) as a promising starting point for further development. Sub-libraries of compounds were designed, synthesized and tested as competitors of these interactions. The testing was performed using SPR technology to detect inhibition of S100A9 binding to RAGE and TLR4 (data not shown). By this method we defined a group of salicylic amides that showed specific binding to S100A9. The best compound in this series was oxyclozanide (OX) that was selected for further studies (Figure 1B).

OX inhibited in a dose-dependent fashion the interaction between S100A9 and both RAGE (Figure 1C) and TLR4 (Figure 1D). To verify that the interaction between OX and S100A9 resembled that which we have previously described for quinoline-3-carboxamides (Q compounds) [21], we also investigated the Ca^++^ and Zn^++^ dependency of the binding interaction of OX and S100A9. As shown in Figure 1E, the interaction between OX and S100A9 was dependent on the presence of both Ca^++^ and Zn^++^ for satiable binding to immobilized S100A9.

**Figure 2:**
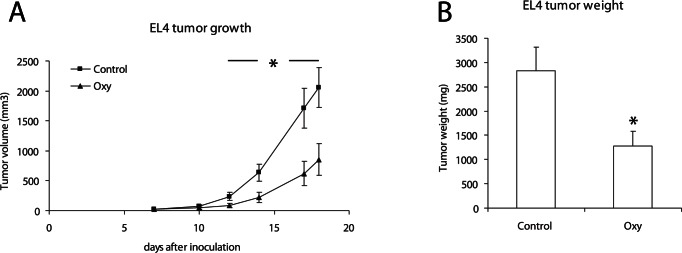
OX inhibits EL4 lymphoma growth *in vivo*. A: Anti-tumor effect of OX in EL4 tumors inoculated (s.c.) into wild type mice. OX was administrated p.o. at 30 mg/kg/day 7 days/week from day 1 throughout the experiment. Each data point represents mean ± SEM (n = 10; *p<0.05, Mann Whitney U). Control animals received only water. B: Tumor weights (*p<0.05; Mann Whitney U).

We have previously also shown that the inhibition of S100A9 and TLR4 expression influenced EL4 tumor growth, and that the inhibition of S100A9 interactions *in vivo* using Q compounds mimicked this effect [24]. Therefore, we performed an experiment where we investigated the effect of OX on EL4 tumor growth. As shown in Figure 2 A and B, OX inhibited EL4 tumor growth both as measured by tumor volume and tumor weight. We conclude from these data that a chemical probe selected only for its ability to bind S100A9 and inhibit its interaction with TLR4 and RAGE, show anti-tumor activity *in vivo*.

### S100A4 and S100A9 Show Common Molecular Interactions

**Figure 3:**
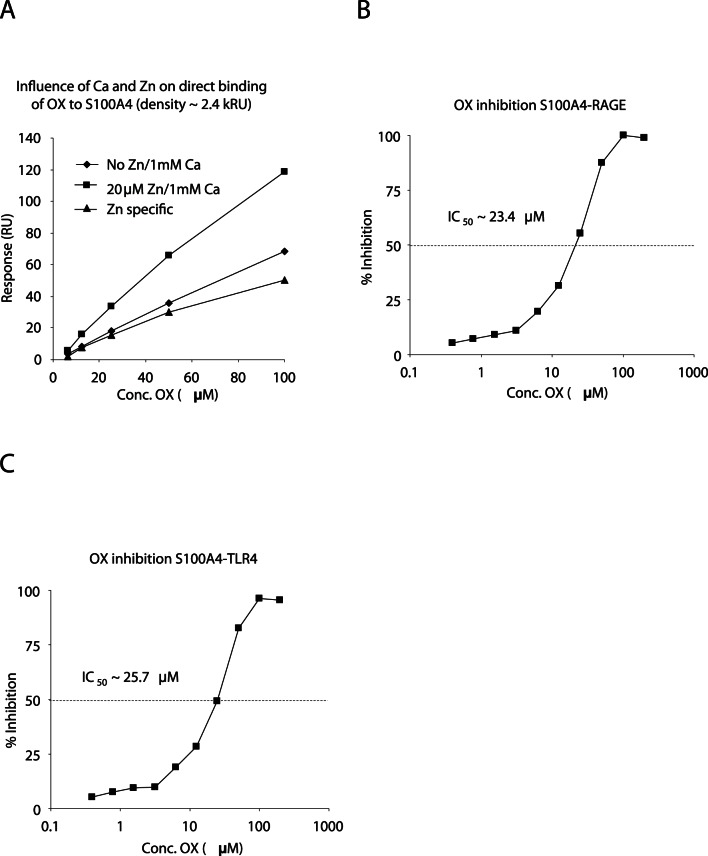
Oxyclozanide binds S100A4. A: Effect of Zn^++^ on direct binding of OX to amine coupled S100A4. 3.125–100 µM OX was injected (2 min at 30 µL/min) over S100A4 (density ∼2.4 kRU) in HBS-P with 1 mM Ca^++^ ±20 µM Zn^++^. Responses at late association phase are plotted versus OX concentration. Zn^++^ specific binding was obtained by subtraction of responses with Ca^++^ alone and curves fit to a 1∶1 model. B-C: Inhibition of S100A4 binding to immobilized RAGE (B) and TLR4 (C) by OX. 100 nM S100A4 was injected over RAGE or TLR4±0.4–200 µM OX in the presence of 1 mM Ca, 20 µM Zn and 1% DMSO. Binding was expressed as % inhibition of response with S100A4 in the absence of OX. IC_50_ values (inserted) were calculated after curve fit to a sigmoidal dose-response model.

Other salicylic amides have previously been described as inhibitors of tumor growth, including niclosamide that has shown anti-tumor activity in models that are dependent on S100A4 [29]. Given the structural similarities between S100 proteins [2], as well as between niclosamide and OX, we proceeded to investigate whether OX could also interact with S100A4. We first investigated whether S100A4 could bind OX directly. As shown in Figure 3A, OX interacts with S100A4 in a similar Ca^++^ and Zn^++^ dependent manner as was described for the binding of OX to S100A9 above. Thus, a very similar conformational epitope, defined by OX binding, must be present in S100A4 and S100A9.

We then proceeded to investigate whether S100A4, in the presence of Ca^++^ and Zn^++^, could also be a ligand of both RAGE and TLR4. Furthermore, we investigated the effect of OX on these interactions. Indeed, a specific interaction between S100A4 and RAGE could easily be demonstrated which could be inhibited by OX although with a ten-fold lower potency compared to the interaction between S100A9 and RAGE (Figure 3B). Very similar findings were observed when the interaction between S100A4 and TLR4 was investigated (Figure 3C). We conclude from these experiments that S100A4 in its Ca^++^/Zn^++^ bound form is a ligand for both RAGE and TLR4.

### S100A4 and S100A9 can form Heterodimers

**Figure 4:**
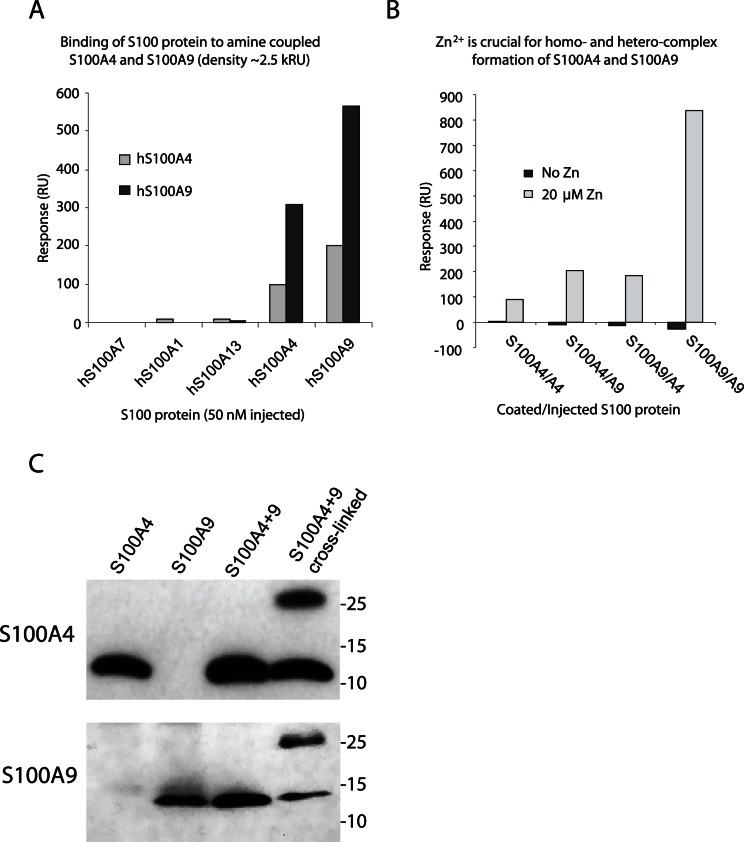
S100A4 and S100A9 can form heterodimers. A: Binding of S100 proteins to immobilized S100A4 and S100A9. S100A1, A4, A7, A9 and A13 (∼1.3 µg/mL) were injected (2 min at 30 µL/min) over S100A4 and S100A9 (density ∼2.5 kRU) in the presence of 1 mM Ca^++^ and 20 µM Zn^++^. Responses were calculated at late association phase. B: Formation of homo- and hetero-complexes of S100A4 and S100A9 is Zn^++^ dependent. S100A4 and S100A9 were injected over immobilized S100A4 or S100A9 at ∼1.3 µg/mL HBS-P containing 1 mM Ca^++^ ±20 µM Zn^++^. Responses at late association phase were calculated. C: HEK293T cells were transfected either with S100A4 or S100A9 cDNA construct alone or the two together, as indicated. After 24 hrs of culture some of the transfected cells were exposed to the membrane permeable cross-linker DSS. Thereafter cell lysates were prepared, equal amounts (30 µg) of protein loaded on an SDS-PAGE gel and western blots were performed using either anti-S100A4 or anti-S100A9 antibodies as indicated. Representative results from one out of two experiments performed are shown.

S100A9 is commonly expressed as a heterodimer together with S100A8 [15], although it can also form homodimers [18]. Given the similarities of S100A9 and S100A4, we wanted to investigate whether S100A9 and S100A4 could interact directly and form heterodimers. To this end we immobilized either S100A4 or S100A9 on a biosensor chip and performed SPR analysis with various S100 proteins in the fluid phase. As shown in Figure 4A, homodimeric interactions were readily detected, independent of whether S100A4 or S100A9 was immobilized, but also interactions between S100A4 and S100A9. No interaction was detected of either protein with S100A1, S100A7 and S100A13 that were included as controls. All complex formation was dependent on Zn^++^ (Figure 4B). Thus, the conformational change induced by Zn^++^ is not only important for the ability of S100 proteins to interact with their ligands, but also seems to affect the formation of S100 protein heterodimers.

To verify that S100A4/S100A9 heterodimers could also be formed *in vivo*, we transfected 293T cells with expression plasmids carrying either human S100A4 or human S100A9 cDNAs. In cells transfected with the S100A4 or S100A9 constructs, S100A4 and S100A9 proteins could be detected. In cells transfected with theS100A9 alone there was very little S100A9 protein detected (Figure 4C). However, in cells transfected with both the S100A4 and the S100A9 constructs the amounts of both proteins were increased, indicating that the two proteins interact. Using a cell-permeable cross-linker, the presence of a protein with the predicted size of a S100A4/S100A9 heterodimer could be revealed, both using S100A4- and S100A9-specific antibodies. These data indicate that, given the appropriate conditions, S100A4/S100A9 heterodimers also form *in vivo*.

**Figure 5:**
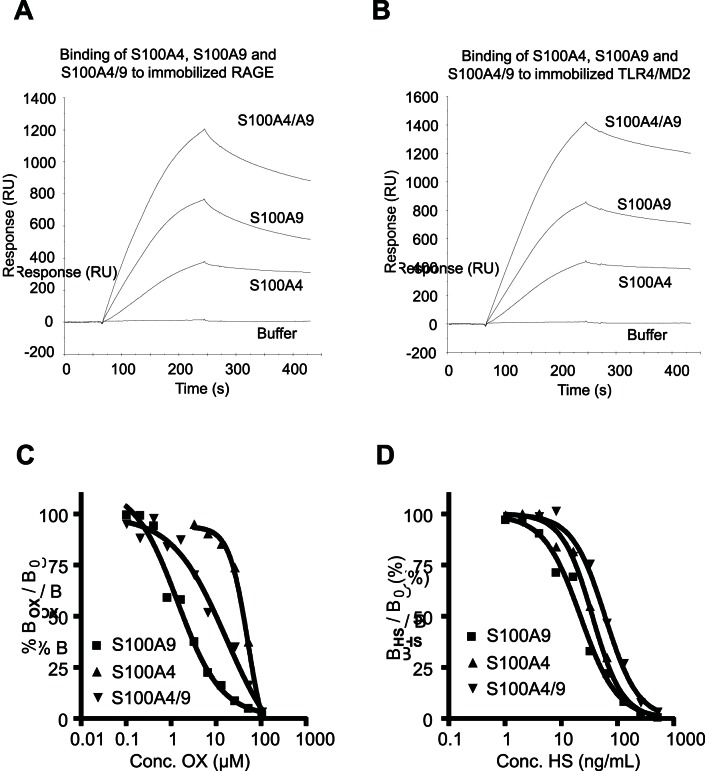
Binding of homo- and hetero-complexes of S100A4 and S100A9 to RAGE are displaced by OX and heparan sulfate (HS). A: Binding of homo- and hetero-complexes of S100A4 and S100A9 to immobilized RAGE and TLR4. S100A4, S100A9 and a 1∶1 mixture of S100A4/9 were injected (2 min at 30 µL/min) over RAGE (left panel) or TLR4 (right panel). B: Inhibition of ∼1.3 µg/mL S100A4, S100A9 and the 1∶1 mixture with 0.195–100 µM OX in the presence of 1 mM Ca^++^, 20 µM Zn^++^ and 1% DMSO. Samples were injected (2 min at 30 µL/min) over immobilized RAGE. C: Responses at late dissociation phase (expressed as % of signal in the absence of OX) were plotted against concentration of competitor and IC_50_ calculated by fit of curves to a sigmoidal dose-response or a one-site competition model. IC_50_ values of 1.4, 52 and 17 µM were calculated for S100A9, S100A4 and S100A4/S100A9. D: Corresponding experiment with 0.98–500 ng HS/mL as competitor. Conditions were identical except that DMSO was omitted from the sample buffer. HS inhibited binding of S100A9, S100A4 and S100A4/S100A9 to RAGE with 50% at 22, 36 and 60 ng/mL.

We next wanted to verify that S100A4/S100A9 heterodimers would be functional, i.e. interact with a relevant receptor. To address this we investigated the ability of S100A4 and S100A9 to interact with RAGE and TLR4 both as homo- and hetero-complexes. As is shown in Figure 5A, S100A4 interacts with RAGE in a similar way as S100A9 but at a lower response level and with a slightly slower off-rate. We could also demonstrate that the S100A4/S100A9 heterodimer binds to RAGE with kinetics resembling that of S100A9 and with a signal that is higher than the combined responses of the respective homodimers. This supports the notion that the heterodimer is biologically relevant and even more since similar results were obtained with TLR4 immobilized on the chip (Figure 5B). When the effect of OX on this interaction was investigated, OX inhibited S100A4/S100A9 binding to RAGE with somewhat lower potency than the S100A9 homodimer but more efficiently than the S100A4 homodimer (Figure 5C). This indicates that similar interfaces are involved when S100A4 and S100A9 interact with RAGE as homo- or hetero-complexes. Lastly, we investigated whether heparan sulfate (HS), a known inhibitor of the S100A9-RAGE interaction, was also able to displace the binding of S100A4 homo- and heterodimers to RAGE. As is shown in Figure 5D, HS inhibited binding of S100A4 homo- and heterocomplexes to RAGE almost as efficiently as S100A9. We conclude from these data that S100A4 and S100A9 show an overlapping reactivity with TLR4 and RAGE and that these proteins can form heterodimers with reactivity to these receptors.

### S100A4 and S100A9 Expression in vivo

**Figure 6:**
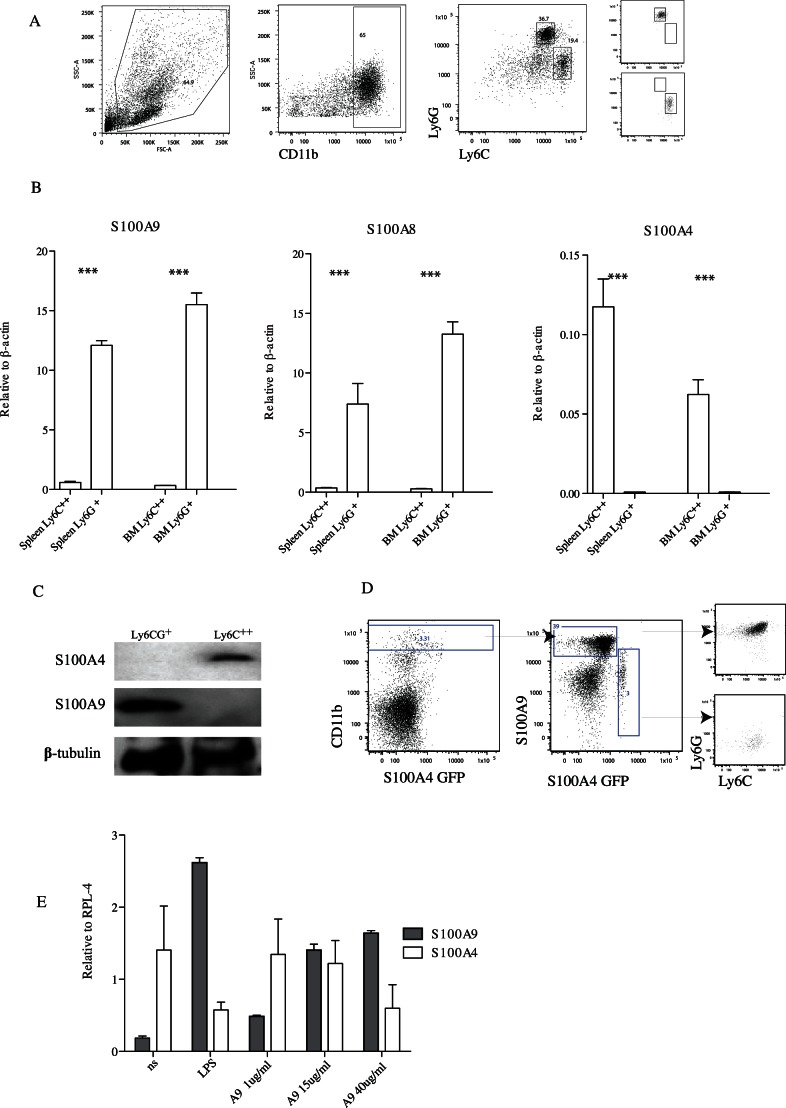
Analysis of S100A9 and S100A4 expression *in vivo*. FACS sorting of spleen cells from C57BL/6 animals. Panel A: Left: The gate used for defining CD11b^+^ cells is shown. Right: The CD11b^+^Ly6G^+^C+/CD11b^+^Ly6C^++^ populations used for comparison and defined as indicated. Purity is shown to the far right. Panel B: Quantitative real time RT-PCR analysis of S100A9, S100A8 and S100A4 RNA expression from indicated cell populations (>90% pure by FACS analysis) from the spleen and BM of C57BL/6, animals. The mean expression from 4 separate experiments is shown (*** = p<0.001; Students unpaired t test). C. FACS Aria sorted cells were used for Western blotting. Antibodies to S100A4, S100A9 and β-tubulin were used to reveal respective protein. Panel D. Indicates the gating strategy on S100A4 GFP mice on C57BL/6 background. E. Quantitative RT-PCR analysis of S100A4 and S100A9 expression from THP-1 cells cultivated alone or for 48 h with LPS or S100A9, as indicated. Two independent experiments with triplicate cultures were performed.

Having noted above that S100A4 and S100A9 can form heterodimers *in vitro* we wanted to investigate the expression pattern of these proteins *in vivo*. CD11b^+^ cells in the mouse can be divided into various subpopulations dependent on their cell surface expression of the Ly6C and Ly6G markers (Figure 6A). We isolated by cell sorting CD11b^+^Ly6C^+^Ly6G^+^ (Ly6G^+^) and CD11b^+^Ly6C^++^Ly6G^−^ (Ly6C^++^) cells from bone marrow and spleen from normal C57BL/6 mice and analyzed these two populations for S100A9, S100A8 and S100A4 RNA expression using Q-PCR. The Ly6G^+^ cell population expressed relatively high levels of S100A8 and S100A9 RNA, but was essentially negative for S100A4 expression (Figure 6B). On the contrary, Ly6C^++^ cells expressed significant levels of S100A4 RNA, but relatively low levels of S100A8 and S100A9 compared to Ly6G^+^ cells. In none of the experiments performed we observed a difference between bone marrow or spleen-derived CD11b^+^ cells.

To further verify the differential expression of S100A9 and S100A4 we sorted the same cell populations as above from C57BL/6 spleen and prepared cell extracts for Western blot analysis. As shown in Figure 6C, the selective S100A4 expression in Ly6C^++^ cells could be confirmed at the protein level. In addition, in splenic CD11b^+^ cell from a mouse heterozygous for an allele where the S100A4 gene has been replaced with green fluorescent protein (GFP) the GFP protein appeared to be specifically expressed in the Ly6C^++^ population (Figure 6D). In contrast, intra-cellular staining with anti-S100A9 antibodies revealed a more pronounced staining in the Ly6G^+^ population (Figure 6D).

We subsequently investigated whether the same differential expression between S100A9 and S100A4 could be observed in a human monocytoid cell line. We therefore analyzed the S100A9 and S100A4 RNA expression in the human monocyte cell line THP-1 (Figure 6E). In these cells the S100A9 RNA expression was relatively low while S100A4 RNA expression was higher. Interestingly, after stimulation with LPS the S100A9 expression was significantly up-regulated while the S100A4 expression was down-regulated, again iterating the pattern of differential expression between the two genes. Also, stimulation of THP-1 cells with S100A9 itself, being a TLR4 ligand [30], also up-regulated the S100A9 expression while it down-regulated S100A4 expression.

### The Selectivity of S100A4 and S100A9 Expression Remains during Disease

**Figure 7:**
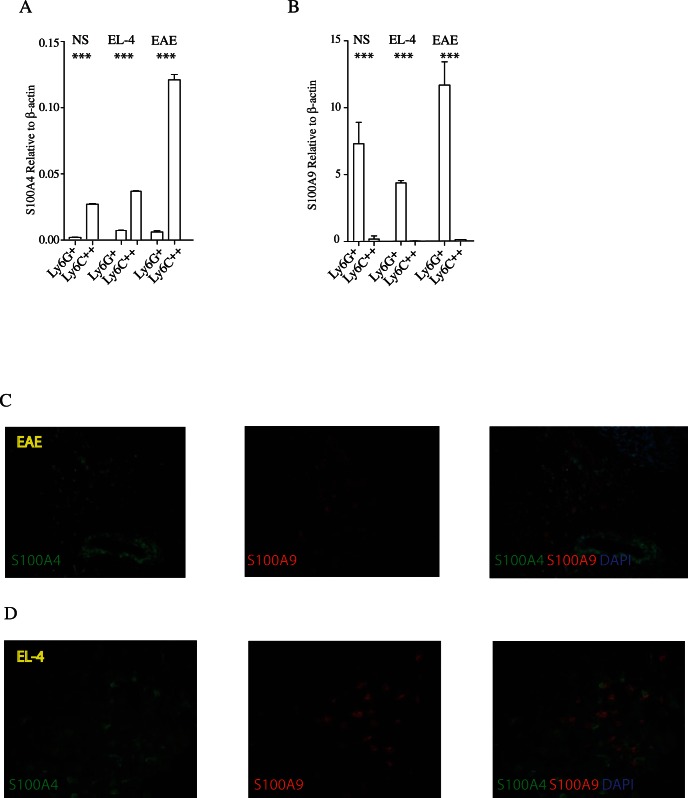
Analysis of S100A9 and S100A4 expression *in vivo* during disease. Panel A and B; Quantitative real time RT-PCR analysis (see Materials and Methods). Spleen cells, from control C57BL/6 animals, from mice inoculated with 50,000 EL4 lymphoma cells or spleen cells from EAE induced animals with MOG-peptide and with Pertussis toxin were FACS-sorted and used to analyze RNA expression of S100A4 (Panel A) and S100A9 (Panel B) (*** = p<0.001; Students unpaired t test). Panel C; Immunohistochemical analysis show cells expressing S100A4 (green), S100A9 (red) in brain sections from mice with MOG induced EAE. The labeled cells are localized close to and around a vessel. An overlay of the two first pictures including nuclear staining of DAPI (blue) is shown in the last picture. Scale bar represents 100 µm. Panel D; Expression of S100A4 (green) and S100A9 (red) respectively in sections from an EL-4 tumor. An overlay of the two first pictures including nuclear staining with DAPI (blue) is shown in the last picture. The overlay shows that S100A4 and S100A9 is expressed in different cells, no overlap can be detected. Scale bar represents 50 µm.

Lastly we wanted to investigate whether the differential expression pattern between S100A4 and S100A9 was modified under disease conditions. We focused on two different disease models; experimental autoimmune encephalitis (EAE) as a model for inflammatory disease and the EL-4 lymphoma model as a model for cancer. First we analyzed the S100A4 and S100A9 RNA expression in splenic Ly6G^+^ and Ly6C^++^ cells in control, EAE and EL-4 inoculated animals. As shown in Figure 7A, S100A4 expression remained largely restricted to the Ly6C^++^ cell population also in disease. Interestingly, while a slight down-regulation of S100A4 expression was observed in EL4 tumor-carrying animals, induction of EAE rather increased the S100A4 RNA expression in splenic Ly6C^++^ cells. A slight increase in S100A9 RNA expression was observed in splenic Ly6C^++^ cells both in EAE and in EL-4 inoculated animals (Figure 7B). An increased S100A9 expression was observed in splenic Ly6C^++^ cells in both EAE and EL-4 inoculated animals. Although these slight differences in expression levels could be seen, we conclude that the differential expression of S100A4 and S100A9 in splenic CD11b^+^ cells remains also during inflammatory disease or tumor challenge.

To confirm this finding we performed immune-histology on brain sections from mice with EAE, as well as on EL4 tumors. As shown in Figure 7C, S100A4 and S100A9 expression could be detected in EAE brain. However, double positive cells were extremely rare. Essentially the same staining pattern was seen in sections from EL-4 tumors (Figure 7D). Most of these cells were also CD11b^+^ (data not shown). We conclude from these data that S100A9 and S100A4 appears to be expressed in distinct CD11b^+^ cell populations *in vivo*. Hence, if the proteins should be able to associate *in vivo* they would most likely have to interact in the extra-cellular space.

## Discussion

In this paper we show that it is possible to define a novel small molecule compound (OX) that has anti-tumor effect *in vivo* by selecting for binding to S100A9 and inhibition of its interaction with RAGE. This finding also indicates that it is quite feasible to find small molecules that can inhibit specific protein-protein interactions. We have previously shown that Q compounds bind to S100A9 and inhibit its interaction with RAGE as well as TLR4 [21]. Also in the case of OX, selection for inhibition of the S100A9/RAGE interaction resulted in a compound that also inhibited the interaction between S100A9 and TLR4. Hence, a very similar molecular structure in S100A9 must be involved in both interactions. Interestingly, this molecular surface is most likely a conformational epitope since binding of S100A9 to OX, Q compounds, RAGE and TLR4 requires the presence of both Ca^++^ and Zn^++^ ([21] and herein). Especially the need for Zn^++^ is intriguing since the levels of free ions needed is unlikely to be available in the intra-cellular environment. Thus, it could be argued that these functions are acquired when exported into the extra-cellular space and that these protein-protein interactions hence are mostly relevant in the extra-cellular compartment.

Q compounds have shown anti-tumor effects *in vivo* [26], [31], [32]. It has also been shown that treatment with Q compounds has a similar effect on EL4 lymphoma growth as seen in animals lacking either S100A9 or TLR4 expression [24]. We show here that OX, which has a very similar effect on S100A9-RAGE/TLR4 interactions as Q compounds, also shows anti-tumor effect *in vivo* in the same tumor model. It could be argued that since OX also interacts with S100A4 (see below), the anti-tumor effect could be due to S100A4 inhibition. However, OX displaces binding of S100A9 more efficiently than binding of S100A4 to both RAGE and TLR4. Moreover, Q compounds do not bind S100A4 (data not shown), which would argue for that, at least in the EL4 lymphoma model, the anti-tumor effect of OX is related to S100A9 binding. Thus, we would like to propose S100A9 as a pertinent target for the development of novel anti-cancer treatments.

The data showing that S100A4 is also able to bind to RAGE, TLR4 and OX was at first glance somewhat surprising. All these interactions were weaker than those between S100A9 and the same molecules but very specific and dependent on the presence of Ca^++^ and Zn^++^, indicating a conformational epitope. It should be noted that the S100 proteins, although diverging at the level of primary sequence, show very similar three-dimensional structures [2]. Our current observation might also indicate that there will be some functional redundancy to be expected between proteins in the S100 protein family. Lastly, the fact that OX interacts with both S100A4 and S100A9 while Q compounds only interacts with S100A9 opens up the possibility for designing S100 protein binding molecules that will display variable patterns of S100 protein interactions.

That S100A4 and S100A9 can form heterodimers (Figure 4) is a novel observation. However, we also note that the formation of these heterodimers is dependent on Zn^++^, which might indicate that such dimers are preferentially formed in the extra-cellular space. The fact that the two proteins seem to be produced by distinct subpopulations of CD11b^+^ cells (Figure 6) would argue for this fact. However, we also show that the heterodimers can be formed intra-cellularly upon forced co-expression of the two proteins (Figure 4C) and it should be noted that S100A9 mRNA is present also in the CD11b^+^ cells that express S100A4, although at low levels. These data indicate that intracellular conditions may still allow dimerisation to stabilize the proteins but that these dimers may not be functional until exported into the extracellular space. Lastly, the S100A4/S100A9 heterodimers are functional with regard to RAGE binding which in turn could be inhibited by OX. The behavior of S100A4/S100A9 heterodimers resembled S100A9 homodimers with regard to binding strength to RAGE, OX inhibition and also that the interaction could be inhibited by heparan sulphate (Figure 5C). However, the S100A4/S100A9 heterocomplex differed from the S100A4 homodimer with regard to the binding strength to both RAGE and TLR4; an effect that seems to be more than additive. This observation could indicate that the secretion of S100A4 in the presence of S100A9 may give rise to multimers with a more potent RAGE and TLR4 stimulatory activity than the secretion of S100A4 alone. In addition, qualitative changes cannot be excluded since both S100A4 and S100A9 can interact with other receptors than RAGE and TLR4 and heterodimerization may create a ligand that can bind additional receptors.

The differential expression of S100A4 and S100A9 in both healthy animals and in animals with ongoing inflammatory disease or tumor burden was striking. In sections from inflamed brain or tumors, double-expressing cells were very rare, if not completely absent. Whether this is of biological significance for the role of S100 proteins in the control of inflammation or tumor growth merits further investigation. An interesting observation was that while S100A4 expression was down-regulated upon inflammatory signals in THP1 cells S100A9 was up-regulated. We believe that this may reflect differentiation of S100A4 expressing THP-1 cells to S100A9 expressing cells. Alternatively, the cell line may contain two populations, one expressing S100A4 and the other expressing S100A9. Upon stimulation, the latter population might outgrow the former. In contrast, the S100A4 expression in Ly6C^++^ monocytes was slightly increased during inflammation *in vivo* while S100A9 expression was substantially increased in the Ly6G^+^ population (Figures 6 and 7). On the contrary, S100A4 was not up-regulated in Ly6C^++^ cells from animals with tumors while S100A9 expression was up-regulated in Ly6G^+^ cells in these animals, in line with what has previously been described for both proteins individually.

In conclusion, we have demonstrated novel interactions between S100 proteins, as well as their interactions with biologically relevant pro-inflammatory receptors. In addition, we believe that this work validates S100A9 as a potential molecular target for the treatment of malignant disease.

## Materials and Methods

### General Synthesis of Salicylic Amides

To a solution of the salicylic acid in dichloromethane were added 4 eq. of oxalylic chloride and one drop of DMF. The reaction mixture was stirred for three hours at room temperature and was then evaporated to dryness, co-evaporated twice with toluene and dried under high vacuum. To a solution of the aniline (2 eq.) and dimethyl aniline (2 eq.) in dioxane (20 ml/gram of acid chloride) at room temperature was added 1 eq. of solid salicylic acid chloride in one portion. The mixture was stirred at room temperature for 2 hours and then 2 M NaOH(aq) (2 eq.) was added, after which stirring was continued for two hours. 5 M sulfuric acid(aq) was added dropwise until pH 0–1 was reached and a precipitate was formed. Water was added to complete the precipitation and the crude product was isolated by filtration. After drying, the crude was purified by crystallization from HOAc/water or EtOH/water. Yields ranged from 20–50%. Oxyclozanide (OX) was purchased from Fluka (99.5%) and used without any further purification.

### Surface Plasmon Resonance (SPR)

SPR analysis was conducted using the Biacore 3000™ system from GE Healthcare, Uppsala, Sweden, mainly as described previously [21]. In the inhibition assay format, recombinant human S100A9 (produced in *E. coli* at Active Biotech; S100A9) or human S100A4 (R&D Systems; S100A4) was injected with OX over human RAGE or TLR4/MD2 immobilized on a CM5 chip at a density of ∼3 kRU. In a first step OX was serially diluted in 100% DMSO and then diluted 50-fold in 10 mM HEPES, 0.15 M NaCl, pH 7.4, containing 0.005% Surfactant P20 (HBS-P buffer). Prior to injection, S100A4 or S100A9 was pre-incubated with OX for ≥1 h in assay buffer (HBS-P containing 1 mM Ca^++^, 20 µM Zn^++^ and 1% v/v DMSO). In order to test the influence of Ca^++^ and Zn^++^ on direct binding of OX to immobilized S100A4 and S100A9, OX was injected in HBS-P without DMSO in the absence or presence of Ca^++^ and/or Zn^++^. The ability of S100A4 and S100A9 to form homo- and hetero-complexes was also tested by injecting S100A4 and S100A9 over these proteins immobilized on the sensor chip and with recombinant human S100A1 (ProSpec, Rehovat, Israel), S100A7 (ProSpec) and S100A13 (R&D Systems) as negative controls. Formed homo- and hetero-complexes were also tested for binding to RAGE and TLR4 and whether binding could be displaced with OX and heparan sulfate (Sigma; HS). Evaluation of binding data was made using GraphPad Prism 4.0 and BIAevalution 3.0 software.

### EAE and EL-4 lymphoma Model

All animal experiments have been approved by a local ethics committee (“Malmö/Lunds Djurförsöksetiska nämnd”). The issued ethical permits DNR M 275-08, M 60-10 and M 04-11 are specifically approved for the experiments performed in the current investigation. C57BL/6 mice, (Taconic M&B, Ry, Denmark) mice were kept in an SPF animal facility at BMC or Active Biotech AB, Lund. Nine weeks old animals were injected subcutaneously with 30,000 EL4 lymphoma cells in 100 µl PBS. After 7 days the animals were scored for tumor growth by palpation, day 10, 12, 14 and 18 the tumors were measured with a slide caliper and the volume calculated. OX was administered by oral gavage 30 mg/kg/daily from the day after cell inoculation to day 17. The mice were sacrificed by cervical dislocation and spleens were dissected. The cell suspension was thereafter passed through a 70 µm cell strainer and cells washed in Hank’s BSS (Invitrogen Life Technologies, Paisley, UK).

### Immunization EAE

Mice were immunized subcutaneously at the base of the tail with 50 µg MOG_35–55_ peptide (Schafer-N, Copenhagen, Denmark) in PBS emulsified in complete Freund’s adjuvant (CFA). To induce the development of EAE, MOG-peptide immunized C57Bl/B6 mice were also injected twice (on the same day and two days later) with Pertussis toxin (200 ng; List Biological Laboratories, Inc. Campbell, Ca) in PBS.

### Cell Culture Conditions

The human monocytic leukaemia cell line THP-1 (purchased from American Type Culture Collection, Manassas, VA) was grown in RPMI-1640 culture medium (Invitrogen, Stockholm, Sweden) supplemented with 10% fetal bovine serum (Invitrogen), 2 mM glutamine (Sigma- Aldrich, St Louis, MO), 1 mM sodium pyruvate, 10 mM HEPES, 100 U/ml penicillin and 100 lg/ml streptomycin (P/S; Invitrogen), at 37° in 5% CO2. The cells were cultured with or without 100 ng/ml ultra pure lipopolysaccharide and various concentrations of S100A9 as indicated in figure for 48 hr followed by harvesting the RNA for Q-PCR. 293T cells were cultured in Dulbecco’s Modified Eagle Medium (DMEM) supplemented with 10% fetal bovine serum (Invitrogen), 2mM Glutamine (Sigma-Aldrich, St. Louis, MO), 100 U/ml penicillin and 100 µg/ml streptomycin (Invitrogen) at 37°C in 5% CO_2_, 1 mM sodium pyruvate, 10 mM HEPES.

### 293T Cell Transfection and Stimulation

Three different transfections were performed: 1) **hS100A4**∶1 µg pcDNA3.1-hS100A4+1 µg pcDNA3.1-EGFP; 2) **hS100A9**∶1 µg pDream2.1-hS100A9+ pcDNA3.1-EGFP; 3) **S100A4/S100A9**∶1 µg pcDNA3.1-hS100A4+1 µg pDream2.1-hS100A9. Briefly, 293T cells were seeded in 24 well plate in serum-free medium. DNA-Lipofectamine 2000 mixture was prepared as follow: 2 µl Lipofectamine 2000/reaction were incubated in 50 µl Optimem medium for 5 min at room temperature (RT). Plasmid DNA was added to Lipofectamine 2000 solution and incubated for further 20 minutes at RT. Subsequently, the DNA-Lipofectamine 2000 mixture was added to the 293T cells, which were incubated 3h at 37°C. Finally, 1 ml/well of full medium was added to the transfected 293T cells and a further 24h incubation at 37°C was performed. In some experiments, to study protein oligomerization, 293T cells were washed in PBS and incubated for 1h in ice with 1 mM disuccinimidyl suberate (DSS) in PBS. Then, 293T cells were washed in PBS, lysed and SDS-PAGE followed by western blot was performed.

### Q-PCR

Splenic CD11b^+^ cells were purified using anti-CD11b magnetic beads and LS-columns (Miltenyi Biotech, Bergisch Gladbach, Germany),. Total RNA was extracted from CD11b^+^ cell preparations by use of the Purelink RNA mini Kit (Invitrogen). RNA was reverse transcribed to cDNA by use of the SuperScript III Platinum synthesis system (Invitrogen). Real-time PCR (RT-PCR) was performed for the detection of S100A9, S100A8 and S100A4 RNA and quantified using a SYBR GreenER kit (Invitrogen) in a MYIQ (Bio-Rad) PCR machine. The threshold cycle number was determined and relative expression level of each mRNA was determined using the formula 2^(Rt– Et)^, where Rt and Et are the threshold cycles for the reference gene (β-actin or RPL-4) and the target gene, respectively.

### Flow Cytometry

Flow cytometry analysis was performed on spleen cell suspensions, as indicated. Primary antibodies used were: anti-mouse CD11b-APC (eBioscience), Ly6G-FITC (BD Pharmingen) and Ly6C-biotin (BD Pharmingen). Biotinylated antibodies were detected with streptavidin-QD605 (Invitrogen). Data were acquired using a FACS LSR II flow cytometer (BD Biosciences) and analyzed using FlowJo software (Tree Star).

### Immunohistochemistry

Tissues analyzed with immunohistochemistry were embedded in OCT compound (Tissue-Tek®), and snap-frozen in liquid nitrogen. Cryosections (7–8 µm) were prepared on microscope slides, air dried and frozen at −20°C until staining procedures. Acetone fixed sections from EL-4 tumors or brains from animals with MOG induced EAE were blocked with 10% normal serum diluted in PBS with 1% BSA for 30 min. Thereafter the sections were incubated with primary antibodies, rabbit anti S100A4 (DAKO Denmark A/S, Denmark) and goat anti S100A9 (Santa Cruz Biotechnology Inc, CA, USA) or isotype controls for each antibody 1 hour in room temperature followed by incubation with secondary antibodies, donkey anti rabbit Alexa Fluor 488 (Invitrogen) and donkey anti goat Alexa Fluor 555 (Invitrogen, Oregon, USA) for 30 min. The slides were mounted using ProLongGold antifade kit containing DAPI (Invitrogen, Oregon, USA). The stainings were analysed and photographs taken using a Leica DMX microscope and Leica application suite 3.7 software.

### Western Blot

Spleen cells were stained as described above and Ly6G^+^ and Ly6C^++^ subpopulations were sorted using a FACSAria flow cytometer (BD Biosciences). For Western blot, 10 µg of proteins was loaded onto 12% polyacrylamide gels (C.B.S. Scientific, San Diego, CA, USA). Proteins were subsequently transferred to PVDF membrane (Roche), which was saturated with 1% dry milk in PBS-Tween. Thereafter, the membranes were incubated with Rat anti-Mouse S100A9, Rabbit anti-mouse S100A4 and Rabbit anti-β Tubulin (R&D Systems) as primary antibody and Rabbit anti-Rat-HRP or Goat anti-Rabbit-HRP (SouthernBiotech Birmingham, Alabama, USA) as secondary antibodies and filters developed using ECL kit (GE Healthcare, UK).

## References

[1] Marenholz I, Heizmann CW, Fritz G (2004) S100 proteins in mouse and man: from evolution to function and pathology (including an update of the nomenclature). Biochem Biophys Res Commun 322: 1111–1122. 15336958 10.1016/j.bbrc.2004.07.096

[2] Heizmann CW, Fritz G, Schafer BW (2002) S100 proteins: structure, functions and pathology. Front Biosci 7: d1356–1368. 11991838 10.2741/A846

[3] Davies BR, Davies MP, Gibbs FE, Barraclough R, Rudland PS (1993) Induction of the metastatic phenotype by transfection of a benign rat mammary epithelial cell line with the gene for p9Ka, a rat calcium-binding protein, but not with the oncogene EJ-ras-1. Oncogene 8: 999–1008. 8455951

[4] Maelandsmo GM, Hovig E, Skrede M, Engebraaten O, Florenes VA, et al (1996) Reversal of the in vivo metastatic phenotype of human tumor cells by an anti-CAPL (mts1) ribozyme. Cancer Res 56: 5490–5498. 8968106

[5] Garrett SC, Varney KM, Weber DJ, Bresnick AR (2006) S100A4, a mediator of metastasis. J Biol Chem 281: 677–680. 16243835 10.1074/jbc.R500017200

[6] Xie R, Schlumbrecht MP, Shipley GL, Xie S, Bassett RL Jr, et al (2009) S100A4 mediates endometrial cancer invasion and is a target of TGF-beta1 signaling. Lab Invest 89: 937–947. 19506550 10.1038/labinvest.2009.52 PMC2718065

[7] Rudland PS, Platt-Higgins A, Renshaw C, West CR, Winstanley JH, et al (2000) Prognostic significance of the metastasis-inducing protein S100A4 (p9Ka) in human breast cancer. Cancer Res 60: 1595–1603. 10749128

[8] Maelandsmo GM, Florenes VA, Nguyen MT, Flatmark K, Davidson B (2009) Different expression and clinical role of S100A4 in serous ovarian carcinoma at different anatomic sites. Tumour Biol 30: 15–25. 19194111 10.1159/000199447

[9] Boye K, Nesland JM, Sandstad B, Maelandsmo GM, Flatmark K (2010) Nuclear S100A4 is a novel prognostic marker in colorectal cancer. Eur J Cancer 46: 2919–2925. 20719498 10.1016/j.ejca.2010.07.013

[10] Yammani RR, Carlson CS, Bresnick AR, Loeser RF (2006) Increase in production of matrix metalloproteinase 13 by human articular chondrocytes due to stimulation with S100A4: Role of the receptor for advanced glycation end products. Arthritis Rheum 54: 2901–2911. 16948116 10.1002/art.22042

[11] Miranda KJ, Loeser RF, Yammani RR Sumoylation and nuclear translocation of S100A4 regulate IL-1beta-mediated production of matrix metalloproteinase-13. J Biol Chem 285: 31517–31524. 10.1074/jbc.M110.125898 PMC2951226 20685652

[12] Kriajevska M, Bronstein IB, Scott DJ, Tarabykina S, Fischer-Larsen M, et al (2000) Metastasis-associated protein Mts1 (S100A4) inhibits CK2-mediated phosphorylation and self-assembly of the heavy chain of nonmuscle myosin. Biochim Biophys Acta 1498: 252–263. 11108967 10.1016/s0167-4889(00)00100-2

[13] Ambartsumian N, Klingelhofer J, Grigorian M, Christensen C, Kriajevska M, et al (2001) The metastasis-associated Mts1(S100A4) protein could act as an angiogenic factor. Oncogene 20: 4685–4695. 11498791 10.1038/sj.onc.1204636

[14] Foell D, Wittkowski H, Vogl T, Roth J (2007) S100 proteins expressed in phagocytes: a novel group of damage-associated molecular pattern molecules. J Leukoc Biol 81: 28–37. 16943388 10.1189/jlb.0306170

[15] Roth J, Vogl T, Sorg C, Sunderkotter C (2003) Phagocyte-specific S100 proteins: a novel group of proinflammatory molecules. Trends Immunol 24: 155–158. 12697438 10.1016/s1471-4906(03)00062-0

[16] Foell D, Roth J (2004) Proinflammatory S100 proteins in arthritis and autoimmune disease. Arthritis Rheum 50: 3762–3771. 15593206 10.1002/art.20631

[17] Hermani A, Hess J, De Servi B, Medunjanin S, Grobholz R, et al (2005) Calcium-binding proteins S100A8 and S100A9 as novel diagnostic markers in human prostate cancer. Clin Cancer Res 11: 5146–5152. 16033829 10.1158/1078-0432.CCR-05-0352

[18] Itou H, Yao M, Fujita I, Watanabe N, Suzuki M, et al (2002) The crystal structure of human MRP14 (S100A9), a Ca(2+)-dependent regulator protein in inflammatory process. J Mol Biol 316: 265–276. 11851337 10.1006/jmbi.2001.5340

[19] Lominadze G, Rane MJ, Merchant M, Cai J, Ward RA, et al (2005) Myeloid-related protein-14 is a p38 MAPK substrate in human neutrophils. J Immunol 174: 7257–7267. 15905572 10.4049/jimmunol.174.11.7257

[20] Manitz MP, Horst B, Seeliger S, Strey A, Skryabin BV, et al (2003) Loss of S100A9 (MRP14) results in reduced interleukin-8-induced CD11b surface expression, a polarized microfilament system, and diminished responsiveness to chemoattractants in vitro. Mol Cell Biol 23: 1034–1043. 12529407 10.1128/MCB.23.3.1034-1043.2003 PMC140712

[21] Bjork P, Bjork A, Vogl T, Stenstrom M, Liberg D, et al (2009) Identification of Human S100A9 as a novel target for treatment of autoimmune disease via binding to quinoline-3-carboxamides. PLoS Biology 7: 800–812. 10.1371/journal.pbio.1000097 PMC2671563 19402754

[22] Gebhardt C, Riehl A, Durchdewald M, Nemeth J, Furstenberger G, et al (2008) RAGE signaling sustains inflammation and promotes tumor development. J Exp Med 205: 275–285. 18208974 10.1084/jem.20070679 PMC2271015

[23] Apetoh L, Ghiringhelli F, Tesniere A, Obeid M, Ortiz C, et al (2007) Toll-like receptor 4-dependent contribution of the immune system to anticancer chemotherapy and radiotherapy. Nat Med 13: 1050–1059. 17704786 10.1038/nm1622

[24] Kallberg E, Vogl T, Liberg D, Olsson A, Bjork P, et al (2012) S100A9 interaction with TLR4 promotes tumor growth. PLoS One 7: e34207. 22470535 10.1371/journal.pone.0034207 PMC3314596

[25] Hiratsuka S, Watanabe A, Aburatani H, Maru Y (2006) Tumour-mediated upregulation of chemoattractants and recruitment of myeloid cells predetermines lung metastasis. Nat Cell Biol 8: 1369–1375. 17128264 10.1038/ncb1507

[26] Jennbacken K, Welen K, Olsson A, Axelsson B, Torngren M, et al (2012) Inhibition of metastasis in a castration resistant prostate cancer model by the quinoline-3-carboxamide tasquinimod (ABR-215050). Prostate 72: 913–924. 22287276 10.1002/pros.21495

[27] Gabrilovich DI, Ostrand-Rosenberg S, Bronte V (2012) Coordinated regulation of myeloid cells by tumours. Nature reviews Immunology 12: 253–268. 10.1038/nri3175 PMC3587148 22437938

[28] Cheng P, Corzo CA, Luetteke N, Yu B, Nagaraj S, et al (2008) Inhibition of dendritic cell differentiation and accumulation of myeloid-derived suppressor cells in cancer is regulated by S100A9 protein. J Exp Med 205: 2235–2249. 18809714 10.1084/jem.20080132 PMC2556797

[29] Jin Y, Lu Z, Ding K, Li J, Du X, et al (2010) Antineoplastic mechanisms of niclosamide in acute myelogenous leukemia stem cells: inactivation of the NF-kappaB pathway and generation of reactive oxygen species. Cancer Res 70: 2516–2527. 20215516 10.1158/0008-5472.CAN-09-3950

[30] Riva M, Kallberg E, Bjork P, Hancz D, Vogl T, et al (2012) Induction of nuclear factor-kappaB responses by the S100A9 protein is Toll-like receptor-4-dependent. Immunology 137: 172–182. 22804476 10.1111/j.1365-2567.2012.03619.x PMC3461398

[31] Isaacs JT, Pili R, Qian DZ, Dalrymple SL, Garrison JB, et al (2006) Identification of ABR-215050 as lead second generation quinoline-3-carboxamide anti-angiogenic agent for the treatment of prostate cancer. Prostate 66: 1768–1778. 16955399 10.1002/pros.20509

[32] Olsson A, Bjork A, Vallon-Christersson J, Isaacs JT, Leanderson T (2010) Tasquinimod (ABR-215050), a quinoline-3-carboxamide anti-angiogenic agent, modulates the expression of thrombospondin-1 in human prostate tumors. Mol Cancer 9: 107. 20470445 10.1186/1476-4598-9-107 PMC2885345

